# Sperm Physiological Response to Female Serum—Potential New Insights into the Reproductive Incompatibility Diagnostics

**DOI:** 10.3390/ijms23073428

**Published:** 2022-03-22

**Authors:** Aleksandra Łukasiewicz, Kari Huhta, Jarmo Ritari, Juha Peräsaari, Pia Allinen, Marjo Malinen, Annalaura Jokiniemi, Tanja Turunen, Jukka Partanen, Jukka Kekäläinen

**Affiliations:** 1 Department of Environmental and Biological Sciences, University of Eastern Finland, P.O. Box 111, 80101 Joensuu, Finland; karhuh@student.uef.fi (K.H.); marjo.malinen@uef.fi (M.M.); annalaura.jokiniemi@uef.fi (A.J.); tanja.turunen@uef.fi (T.T.); jukka.s.kekalainen@uef.fi (J.K.); 2 Research and Development, Finnish Red Cross Blood Service, Haartmaninkatu 8, 00290 Helsinki, Finland; jarmo.ritari@veripalvelu.fi (J.R.); jukka.partanen@veripalvelu.fi (J.P.); 3 Clinical Laboratory, Finnish Red Cross Blood Service, Kivihaantie 7, 00310 Helsinki, Finland; juha.perasaari@veripalvelu.fi; 4 InOva klinikka Oy, Ajurinkatu 16, 70110 Kuopio, Finland; pia.allinen@inova.fi

**Keywords:** sperm function, sexual selection, genetic compatibility, MHC, cryptic female choice, infertility, egg donor, fertilization

## Abstract

Infertility is assumed to arise exclusively from male- and female-dependent pathological factors. However, recent studies have indicated that reproductive failure may also result from the reproductive incompatibility of the partners. Selection against such incompatibilities likely occurs via female-derived reproductive secretions, including follicular fluid (FF), that mediate gamete-level mate choice towards the sperm of specific males. To facilitate potential development of diagnostic tests for human reproductive incompatibility, we examined whether sperm physiological response to female serum indicate male–female compatibility in the presence of FF. We performed a full-factorial experiment, in which the sperm of 10 males were treated with the FF and serum of 6 healthy females. We found that sperm motility and viability in both biofluids were highly similar and that in 70% of the males, sperm serum treatment predicted male–female compatibility. We also identified male human leucocyte antigen (HLA) alleles and female (FF and serum) anti-HLA antibodies and tested whether the number of allele–antibody matches predict sperm physiological response to female fluids. However, no association was found between measured sperm traits and the number of allele–antibody matches. Overall, the present results may open novel possibilities for the future development of reproductive incompatibility tests and may pave the way towards more accurate infertility diagnostics and treatments.

## 1. Introduction

Fertilization success is strongly dependent on gamete-level biochemical interactions occurring between the mating partners [1]. This complex communication process is driven by various gamete surface molecules and female-derived reproductive secretions (e.g., [2,3]). These female-derived signals induce a cascade of changes in sperm physiology, including functional maturation (capacitation), hyperactivation, and acrosome reaction [4,5,6,7,8], and they shape sperm behaviour by stimulating or inhibiting sperm motility [6]. It has been assumed that the primary function of this gamete-level signalling is to ensure that only a few out of millions of spermatozoa can reach the fertilization site and to prevent fertilization-incompetent sperm from reaching the oocytes (e.g., [1,2,9,10]). However, recent studies have indicated that female-induced sperm selection is far from a random process and that it frequently biases paternity towards particular males over others [11,12,13,14]. Female-derived reproductive secretions have therefore additional roles in mediating mate choice at the level of the gametes (gamete-mediated mate choice, GMMC) [1].

Previous work has demonstrated that the highly polymorphic major histocompatibility complex (MHC) immune gene family likely plays an important role in GMMC [15,16,17]. Since possessing a wide diversity of MHC proteins is crucial for efficient immune responses against various infections, GMMC (and mate choice in general) is expected to favour MHC-dissimilar partners, which should enhance the immunocompetence of the offspring (reviewed in [18,19]). Recent studies have indicated that MHC-associated GMMC also occurs in humans [20,21]. These studies showed that sperm physiological response to follicular fluid and cervical mucus of different females is strongly dependent on male–female combination. Furthermore, in support of the above-mentioned MHC-dissimilarity preferences, sperm performance in these female reproductive secretions has been found to be higher in human leukocyte antigen (HLA) dissimilar male–female combinations. Therefore, one of the primary functions of GMMC in both animals and humans may be to facilitate assortative fertilization between genetically compatible mates [22]. Such compatibility differences may have major impact both on the fertilization success and the probability of achieving successful pregnancy.

Human infertility in Western countries is increasing at an alarming rate (e.g., [23]), but we do not understand the mechanistic bases of this decline (e.g., [24]). According to the World Health Organization (WHO), infertility is caused by male- or female-dependent pathological factors. However, this may be an overly simplistic view, as this definition of infertility does not factor in the possibility that infertility may also result from gamete-level incompatibility between the reproductive partners [22]. Kekäläinen [22] also argues that by investigating sperm physiological response to female reproductive tract secretions, we may gain important information that can facilitate development of more realistic functional tests for sperm fertilization capability and reproductive compatibility of the partners (see also [25]). However, given that acquiring female reproductive fluids, such as follicular fluid, requires invasive transvaginal follicular puncture and potentially harmful hormonal treatments, follicular fluid is not readily available for such diagnostic tests.

It has been demonstrated that sperm functional response to female reproductive fluids may be predictable by means of ‘non-reproductive’ biological fluids, such as serum [26,27,28]. Considering that follicular fluid is filtrated from serum in the thecal capillaries of the ovary [29], serum could represent an ideal surrogate for follicular fluid for the development of reproductive compatibility tests. Supporting this view, it has been demonstrated that the human serum hyperactivates sperm cells with as comparable efficiency as follicular fluid [30]. It has also been shown that despite some differences in their biochemical composition [31,32,33], both fluids are similar in their protein, metabolite, ionic, hormone (thyroid and leptin), and immunoglobulin content [32,34,35,36,37,38,39,40]. However, to best of our knowledge, none of the earlier studies have tested whether sperm physiological response to serum could indicate the reproductive compatibility of the partners.

Earlier studies have demonstrated that immunoglobulin G and A antibodies penetrate the blood–follicle barrier [31,35]. Thus, serum and follicular fluid share similar IgG and IgA content. Interestingly, among all immunological factors that may affect fertilization and reproductive success, little attention has been paid to antibodies (but see, e.g., antisperm antibodies studies; [41]), especially to anti-HLA antibodies [42]. Anti-HLA antibodies are formed in response to contact with non-self HLA antigens after blood transfusion, organ or tissue transplantation, and pregnancy. The presence of female anti-HLA antibodies is commonly assumed to be harmless during pregnancy, but they may, however, reduce the probability of live birth in patients with recurrent miscarriages [43]. Given that HLA molecules have been suggested to be expressed also on the surface of human sperm [44,45,46,47], it is possible that follicular fluid anti-HLA antibodies targeted against these sperm surface HLAs could play a role in determining the immunological compatibility of the mating partners.

In the present study, we investigated the possibility of using serum as a surrogate of follicular fluid in evaluating the gamete-level compatibility of the reproductive partners. By using a full-factorial design, we treated the sperm of 10 men with the follicular fluid and serum of 6 women in all possible combinations, resulting two identical 10 × 6 ‘full-factorial blocks’. Then we measured three standard sperm traits: motility, hyperactivation, and viability in all these combinations. We identified follicular fluid and serum anti-HLA (IgG) antibodies and genotyped males for their HLA class I and II alleles. Finally, we tested whether the female anti-HLA antibodies against their target HLA alleles in males (hereafter called as allele–antibody match) predicts the above-mentioned sperm traits after follicular fluid and serum treatment of the sperm.

## 2. Results

### 2.1. Effect of Follicular Fluid and Serum on Sperm Motility and Viability

**Figure 1:**
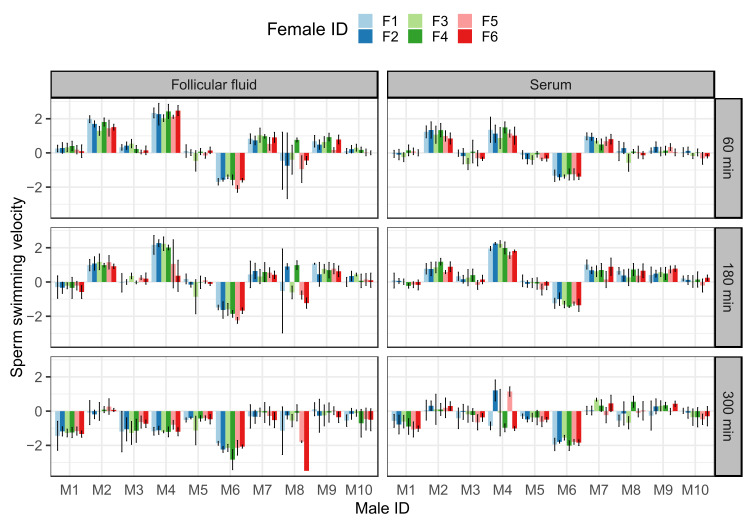
The effect of male:female interaction (combination) on the sperm swimming velocity (calculated on four replicate measurements per combination ± s.e.) at three different time points (60, 180, and 300 min) after the initiation of the follicular fluid or serum treatment. The data were standardized to a mean of 0 and a standard deviation of 1.

The effect of sperm treatment on sperm swimming velocity (VCL) and proportion of hyperactivated sperm cells (HA) varied across time points (treatment × time point interaction: VCL, F = 4.461, *p* = 0.025; HA, χ^2^ = 15.001, *p* < 0.001; Table S1). However, sperm VCL and HA sperm cells did not differ significantly between sperm treatments in any of the three time points (VCL 60 min: F = 0.425, *p* = 0.522; VCL 180 min: F = 0.148, *p* = 0.705; VCL 300 min: F = 2.702, *p* = 0.118; HA 60 min: χ^2^ = 0.635, *p* = 0.426; HA 180 min: χ^2^ = 0.191, *p* = 0.662; HA 300 min: χ^2^ = 1.798, *p* = 0.180; Tables S2 and S3). For both VCL and sperm hyperactivation data, the three-way interaction between treatment, male, and female was significant at 180 and 300 min (VCL 180 min: χ^2^ = 18.372, *p* < 0.001; VCL 300 min: χ^2^ = 45.698, *p* < 0.001; HA 180 min: χ^2^ = 13.182, *p* < 0.001; HA 300 min: χ^2^ = 24.642, *p* < 0.001) but not at 60 min (VCL: χ^2^ = 1.345, *p* = 0.246; HA: χ^2^ = 1.456, *p* = 0.228; Tables S2 and S3). In other words, the effect of male–female combination on sperm motility differed significantly between sperm treatments at the last two time points, but not after 60 min. Treatment-specific analysis revealed that the compatibility effect was delayed in serum treatment: both VCL and hyperactivation were affected by male–female combination after 180 min incubation in the follicular fluid (VCL: χ^2^ = 11.528, *p* < 0.001; HA: χ^2^ = 19.468, *p* < 0.001), but only after 300 min in serum (VCL: χ^2^ = 47.970, *p* < 0.001; HA: χ^2^ = 20.574, *p* < 0.001; Table 1 and Table S4, Figure 1 and Figure S1).

Sperm viability was significantly lower in follicular fluid than in serum treatment (χ^2^ = 5.044, *p* = 0.025; Table S5, Figure S2) and was also affected by the treatment:male:female interaction (χ^2^ = 30.083, *p* < 0.001; Table S5). Treatment-specific models revealed that the male:female interaction effect on sperm viability was significant in the follicular fluid but not in the serum (follicular fluid: χ^2^ = 23.07, *p* < 0.001; serum: χ^2^ = 2.778, *p* = 0.096; Table S6; Figure S3).

### 2.2. Serum Treatment Predicts Sperm Performance in Follicular Fluid

Sperm parameters measured after serum treatment predicted respective sperm traits in follicular fluid treatment at all time points except hyperactivation at 60 min, but also this association was marginally significant (VCL 60 min: F = 5.391, *p* = 0.021, VCL 180 min: F = 9.402, *p* = 0.003, VCL 300 min: F = 9.804, *p* = 0.002; HA 60 min: χ^2^ = 3.388, *p* = 0.066; HA 180 min: χ^2^ = 5.802, *p* = 0.016; HA 300 min: χ^2^ = 13.225, *p* < 0.001, Table 2 and Table S7). A similar association between sperm treatments was found also for sperm viability (χ^2^ = 5.696, *p* = 0.017; Table S8).

At time points at which statistically significant male:female interaction effects were detected (180 min in follicular fluid and 300 min in serum; Table 1 and Table S4), we also found that the effect of male–female combination on sperm VCL and hyperactivation differed significantly between treatments, which was indicated by the significant treatment:male:female interaction (VCL: χ^2^ = 49.487, *p* < 0.001, HA: χ^2^ = 44.413, *p* < 0.001; Table 3). This suggests that the male–female compatibility effect on the sperm traits is not consistent between follicular fluid and serum treatment. However, this inconsistency was driven by 3 out of 10 males. Accordingly, male-specific models (equivalent models fitted for each male separately) uncovered a significant interaction between treatment and female identity only for male 4 (VCL: χ^2^ = 18.879, *p* < 0.001; HA: χ^2^ = 11.957, *p* < 0.001), male 7 (VCL: χ^2^ = 6.162, *p* = 0.013; HA: χ^2^ = 14.701, *p* < 0.001), and male 8 (VCL: χ^2^ = 4.438, *p* = 0.035: HA: χ^2^ = 17.651, *p* < 0.001), but not for rest of the male subjects (*p* > 0.05). After excluding these three males from the analysis, the treatment:male:female interaction was no longer significant (VCL: χ^2^ = 2.839, *p* = 0.092, HA: χ^2^ = 2.297, *p* = 0.130).

### 2.3. Effect of Allele–Antibody Matches on Sperm Performance

Number of detected allele-specific anti-HLA antibodies in female fluids varied between 0 and 43 in follicular fluid (mean 16.83 ± 0.66 s.e.) and between 3 and 53 in serum (mean 21.33 ± 0.76 s.e.). Among all male subjects, 10–16 (mean 14.3 ± 0.58 s.e.) different HLA alleles per male were detected. Total number of observed allele–antibody matches varied between 0 and 6 in follicular fluid and between 0 and 8 in serum treatment. None of the studied sperm traits were affected by the number of allele–antibody matches (VCL: t = 1.168, *p* = 0.248, HA: z = 0.653, *p* = 0.514, viability: z = −0.475, *p* = 0.635).

## 3. Discussion

The chemical communication between gametes allows females not only to guide the sperm cells towards the eggs but also to discriminate individual mating partners based on their genetic compatibility [1,22]. It has recently been demonstrated that gamete-mediated mate choice towards genetically compatible partners likely occurs in humans [20,21,48]. Supporting these earlier findings, our present results show that the effect of both follicular fluid and serum on sperm swimming velocity and hyperactivation were strongly dependent on male–female combination (compatibility), although in serum, male–female compatibility differences manifested later than in follicular fluid. We also found a strong association between the sperm treatments, indicating that the physiological response of sperm to serum predicts sperm performance in the follicular fluid. However, the magnitude of the male–female combination effect varied between treatments, indicating that male–female compatibility may not be directly predictable from sperm serum treatments. On the other hand, this lack of consistency was predominantly caused by 3 out of 10 males, whereas in 70% of the males, the male–female compatibility effect was similar in both fluids. Together, these results indicate that sperm serum treatment may offer novel possibilities for the development of biologically more realistic functional tests for sperm fertilization capability (see [22]). However, further studies in larger groups of individuals are required to better understand the potential diagnostic value of sperm serum treatments.

According to WHO, infertility affects ca. 15% of reproductive-aged couples worldwide and is thought to arise from male- and female-dependent pathological factors, or a sum of sex-specific factors [49]. However, diagnosing infertility is extremely challenging, and in up to 40% of cases, infertility remains unexplained [50]. Recent studies have shown that, in addition to pathological conditions, fertilization failure may also frequently result from gamete-level incompatibility of the reproductive partners [20,21,22,48,51]. Together these earlier findings indicate that development of clinical tests for incompatibility could have great potential in improving the accuracy of infertility diagnostics and thus facilitating the development of more personalized infertility treatments [22]. Here, we investigated the diagnostic value of serum as a potential diagnostic tool for such tests.

Our results demonstrate that sperm physiological response in both biofluids is highly consistent. This indicates that serum and follicular fluid may contain largely similar composition of functionally important chemical factors, such as sperm chemoattractants [2], to differentially regulate sperm pre-fertilization physiology. It must be noted, however, that the concentration of many other substances may differ between serum and follicular fluid due to permeability and selectivity properties of the blood–follicle barrier and secretion activity of the cumulus and granulosa cells [52,53]. These differences might at least partly explain the lack of between-treatment consistency of the male–female combination effect in 30% of studied males. For example, it has been demonstrated that progesterone concentration can vary between follicular fluid and serum [54], which may cause variation in sperm behavioural responses in different fluids [55]. Nevertheless, more detailed biochemical characterisation of both fluids is needed for a deeper understanding of the molecular mechanisms behind these findings. Furthermore, although logistically and ethically challenging in humans, it would also be important to investigate the clinical relevance of our findings by studying the predictive value of sperm follicular fluid and serum treatments by experimentally fertilizing the oocytes after such treatments. Suggestively, previous studies have demonstrated that at least sperm physiological response to follicular fluid can predict both fertilization success and success rates of infertility treatments [54,56].

It has been demonstrated that the immune system may play a particularly important role in gamete-mediated mate choice (reviewed by [1]). However, the exact mechanisms by which female immune systems could drive post-copulatory mating preferences have remained unclear [15,57]. Since it has been suggested that HLA molecules are expressing on the surface of mature sperm [46], it is possible that sperm HLA proteins play some role in facilitating these mating preferences. Although this finding and its interpretation remain controversial (e.g., [58]), this raise the possibility that female immune system might be able to detect male (sperm) HLA genotype based on these cell surface markers. However, it is unclear whether (and how) follicular fluid anti-HLA antibodies could mediate this selection. In any case, since none of the measured sperm traits were associated with the number of male HLA allele and follicular fluid anti-HLA antibody matches, this conundrum needs to be clarified in future studies.

In conclusion, we found that sperm physiological response to both follicular fluid and serum is highly similar and that sperm serum treatments may reveal novel information on the reproductive compatibility of the males and females. Together, these findings may open possibilities for the future development of clinical tests for gamete-level incompatibility. However, more studies on the molecular mechanisms of female-mediated sperm selection and the biochemical composition of both follicular fluid and serum are required before the diagnostic value of the sperm serum treatments in infertility diagnostics can be reliably evaluated. These studies should also pay more attention to testing the robustness of our findings in larger number of male–female combinations. Finally, besides having potentially important clinical implications, our results also show that in addition to females undergoing assisted reproductive treatments [21,48], gamete-mediated mate choice may occur also among healthy females. This in turn suggests that the cryptic female choice may be more ubiquitous among humans than has previously been thought.

## 4. Materials and Methods

### 4.1. Study Subject and Sample Collection

We obtained follicular fluid from healthy egg donors (n = 6 females) enrolled from the private fertility clinic. All the females were Caucasian, their mean age was 25.3 (±3.9 s.e.) years, and their average body mass index was 26.1 (±1.62 s.e.). None of the females smoked tobacco, and four of them had biological offspring. Follicular fluid samples were collected under local anaesthesia by a transvaginal follicular puncture. Prior to collection, follicle maturation was hyperstimulated with follicle-stimulating hormone, and premature ovulation was prevented using a gonadotrophin-releasing hormone antagonist. When the diameter of the largest follicle reached 18–20 mm, human chorion gonadotrophin was administered. After collection, follicular fluid samples were centrifuged at 500× *g* for 10 min, and the supernatant was aliquoted and stored in liquid nitrogen for later use (see below). Blood samples of the female subjects were taken on the same day, just before the follicular fluid collection. These samples were incubated at room temperature for 1 h before centrifugation in 2500× *g* for 10 min. Supernatant containing the serum was separated and stored under identical conditions as follicular fluids.

Male participants (random representatives of the university community; n = 10) voluntarily provided semen samples by masturbation after 2–5 days of sexual abstinence. All the participants were Caucasian, their mean age was 27.2 (±4.6 s.e.) years, and none of them smoked tobacco. All samples fulfilled WHO’s semen quality criteria for cell count (concentration > 15 million/mL), motility (motile cells > 40%), and viability (live cells > 58%) [59]. Semen samples were allowed to liquefy for 30 min at 37 °C, and mature spermatozoa were then separated from seminal plasma by two-layer density gradient centrifugation (PureSperm^®^ 40 and 80, Nidacon International AB, Mölndal, Sweden) according to manufacturer’s instructions. Sperm pellets were resuspended in PureSperm^®^ Wash solution (Nidacon) to final concentration of ca. 50 million cells/mL. All the male participants also provided EDTA blood samples, which were centrifuged at 10,000× *g* for 10 min. Plasma was then removed, and remaining blood was stored in −80 °C until DNA extraction.

### 4.2. Sperm Activation and Measurements for Sperm Performance

Both follicular fluid (FF) and serum (S) samples of each woman were thawed and aliquoted into two independent replicate tubes (A and B, 24 samples in total) and re-frozen in liquid nitrogen. Twenty microliters of previously washed sperm solution from each man was combined 1:1 (volume/volume) with the follicular fluid and serum samples of all the women in all possible male–female combinations (10 males × 6 females). This yielded 120 samples in total (60 combinations × 2 replicate tubes) per each treatment. All the samples were kept at 37 °C during the entire experiment. All the sperm measurements were performed by using fresh sperm on the day of semen collection.

To measure the effect of female fluids on sperm motility, 1 µL of each FF- and S-treated sperm samples was added to pre-warmed (+37 °C) Leja 4-chamber microscope slides (Leja, Nieuw-Vennep, The Netherlands; chamber height 20 µm). Sperm motility measurements (curvilinear velocity: VCL; linearity of the swimming trajectory: LIN; and amplitude of the lateral head displacement: ALH) were performed using computer-assisted sperm analysis (CASA; Integrated Semen Analysis System, ISAS, v. 1.2, Proiser, Valencia, Spain), with a negative phase-contrast microscope (100× magnification) and a capture rate of 100 frames s^−1^. Measurements were performed at three time points: 60 min, 180 min, and 300 min since the beginning of each sperm treatment. The hyperactivated state of the sperm at each time point was determined based on the following three CASA parameters: VCL > 150 µm s^−1^, LIN < 50%, and ALH > 2.0 [60]. Sperm motility was measured for 1477 ± 19 and 1487 ± 16 sperm (mean ± s.e.) sperm cells per male–female combination in FF and S treatment, respectively.

After the last (300 min) motility measurement, 25 µL of each sperm sample was stained with propidium iodide (PI, 5 µg mL^−1^) and incubated in dark for three minutes. Then, 0.5 µL of 1% formalin was added to immobilize the sperm. The volume of 10 µL of PI-treated sperm samples was individually measured with LUNA-FL™ Dual Fluorescence Cell Counter (Logos Biosystems, Annandale, VA, USA), and the number of dead and alive sperm cells was counted. Sperm viability was measured for 19,441 ± 381 and 18,894 ± 371 (mean ± s.e.) per male–female combination in FF and S treatment, respectively.

All the sperm motility and viability measurements included two independent measurements within both replicate tubes, in each of the 60 male–female combinations, resulting in 240 (60 combinations × 2 replicated tube × 2 replicated measurement) recordings per treatment. To minimize a potential time effect on the measured traits, both the beginning of treatments and subsequent measurements in the first replicate tubes (A) were conducted in the following order: FF1, S1, FF2, S2, …, FF6, S6, whereas in the second replicate tubes (B) the order reversed, i.e., S6, …, FF1. This way, each sample was measured after an identical interval from the beginning of the treatment.

### 4.3. Male HLA Genotyping and Test for Female HLA Antibodies

DNA of all male subjects was extracted from EDTA blood using a PureLink^®^ Genomic DNA Kit (Invitrogen™, (Invitrogen, Carlsbad, ON, Canada), according to the manufacturer’s instructions. Male DNA samples were genotyped with an Illumina Global Screening Array-24 v3.0 kit at the Institute for Molecular Medicine Finland (FIMM). Alleles of the HLA genes of class I (A, B, C) and class II (DPB1, DQA1, DQB1, DRB1, DRB3, DRB4, and DRB5) were imputed at four-digit (i.e., protein-level) resolution using a Finnish reference panel [61] and R library HIBAG v1.26.0 [62].

Female follicular fluid and serum samples were tested for allelic level HLA class I and class II IgG antibodies. Antibodies were screened and identified using bead-based multiplex assay performed on the Luminex platform (LABScreen^®^, One Lambda, West Hills, CA, USA). Antibody specificities were identified with single antigen beads with a positive cut-off threshold of the baseline normalized value 1000 mean fluorescence intensity (MFI). All follicular fluid and serum samples were pre-treated with EDTA to prevent the blocking effect of the complement from the assay. Detected antibodies were assigned with HLA Fusion™ software. Antibodies were recorded according to high resolution (i.e., four digits) HLA typing of the beads.

### 4.4. Statistical Analysis

#### 4.4.1. Models Testing for the Effect of Time Point and Sperm Treatments on Measured Sperm Traits

We first used linear mixed models (LMM) to test the effects of follicular fluid and serum on sperm swimming velocity (VCL). To standardise variability between sperm treatments, data were scaled to have a mean of 0 with a standard deviation of 1 prior to analysis. In order to test the effect of time point and sperm treatments on VCL, the full model included fixed effects of time point, sperm treatment, and replicate tube as well as the interaction between time point and sperm treatment. The effect of sperm treatment on VCL in different males, females and male–female combinations was modelled by adding treatment:male, treatment:female, and treatment:male:female interactions as random factors. In order to take into account repeated measures of sperm motility over time, the final random effect terms for these three interactions also included a random slope for time point (i.e., the final terms were: time point|treatment:male, time point|treatment:female, time point|treatment:male:female). A final model also included random slope of time point for the repeated measures of sperm within each tube (time point|sample).

An equivalent full model was fitted for hyperactivation data (proportion of hyperactivated sperm cells) with generalized linear mixed models (GLMM) with a binomial distribution. The number of hyperactivated sperm cells and the total number of observed cells were implemented as two-vector response variables [63]. Viability data (proportion of dead sperm cells) were analysed with a similar binomial GLMM model. However, since viability was measured only once at the end of the experiment (see above), this model only included the main effects of replicate tube and sperm treatment (fixed effects), as well as random effects for treatment:male, treatment:female and treatment:male:female.

#### 4.4.2. Time Point- and Sperm Treatment-Specific Models

As the interaction between time point and treatment was statistically significant for VCL and hyperactivation (see Results), we conducted similar analyses separately for each time point (henceforth ‘time point-specific models’). These models included replicate tube and sperm treatment as fixed effects and sample, treatment:male, treatment:female, and treatment:male:female interactions as random effects. This specification allowed us to test whether the effect of sperm treatment varied across males, females, and male–female combinations in each of the three time points. Furthermore, to test whether the sperm traits were affected by male, female, and male:female interaction within each treatment, we also performed sperm treatment-specific models for VCL, hyperactivation, and viability. These models included replicate tube as a fixed effect, and male, female, and male–female combination as random effects.

#### 4.4.3. Consistency of Gamete Compatibility Effect and Associations between Sperm Treatments

We next tested whether the observed statistically significant male:female interaction (i.e., ‘gamete compatibility’) effect on sperm VCL and hyperactivation was consistent between different sperm treatments. To accomplish this, we modelled interactions between sperm treatments and male–female combination (1|treatment:male:female) for those time points in which significant male:female interaction effects were observed (i.e., 180 min for follicular fluid and 300 min for serum; see Results).

We further tested the association between sperm treatments separately for each time point by including each sperm trait (VCL, hyperactivation and viability) observed in follicular fluid as a response variable and the same sperm trait measured in serum as a fixed covariate. In other respects, these models were similar to the time point-specific models described above.

#### 4.4.4. Effect of Allele–Antibody Matches on Sperm Traits

Finally, we tested the effect of female (follicular fluid and serum) anti-HLA antibodies on measured sperm traits. First, we counted the number of male HLA allele-female antibody matches—i.e., the number of total anti-HLA antibodies present in follicular fluid and serum samples that matched with their target (male) class I and II HLA alleles in each male–female combination. Initially, the total number of allele–antibody matches was included into full models for VCL and hyperactivation (see above) as a three-way interaction (fixed effect) with sperm treatment and time point (in sperm viability models, we included this as two-way interaction with sperm treatment only). Models with the allele–antibody matches were simplified by removing statistically non-significant interactions, with additional assessment of the goodness of fit of simplified models using the Akaike information criterion (AIC).

#### 4.4.5. Fitting, Comparison, and Diagnostics of the Models

Statistical analyses were performed in R, version 4.0.5. LMMs were fitted with the lmer function from the *lme4* package (v1.1.27.1) [64]. The normality of error distribution of all LMMs were graphically verified using Q–Q plots and residual plots. All reported *p*-values for both fixed and random effects in all LMMs were computed with the anova function (package *lmerTest* v3.1.3), based on Type III sum of squares with Satterthwaite’s method, and ranova function (*lmerTest*), respectively [65]. All GLMMs were implemented with overdispersion accounted for by either observation-level random effects (OLRE; glmer function, *lme4* package, v1.1.27.1) [64] or with a beta-binomial distribution (glmmTMB function, *glmmTMB* package, v1.0.2.1) [66,67]. As suggested by Harrison [67], we also compared beta-binomial models with OLRE models to assess the reliability of each approach; the best-fitted models according to AIC values are reported. We calculated coefficient of determination values (R^2^, i.e., proportion of variance explained) for our models according to Nakagawa and Schielzeth [68]. R^2^ values were calculated separately for fixed factors (marginal R^2^) and for both fixed and random factors (conditional R^2^). The significance of single parameters of fixed effects in all GLMMs was determined with the *car*::Anova function, with type III Wald chi-square tests; significance of single parameters of random effects were determined with likelihood ratio tests (R’s base anova function), by comparing reduced models against full models [69].

## Figures and Tables

**Table 1:** Treatment-specific linear mixed models (LMM) for sperm swimming velocity (VCL) in each of the three time points. Models included replicate tube as fixed effect and male, female, male:female interaction, and repeated measurement of each tube (sample) as random effects. R^2^m: marginal R^2^—proportion of variance explained by fixed factors; R^2^c: conditional R^2^—proportion of variance explained by fixed and random factors.

Time Point	60 Min						180 Min						300 Min					
Treatment	Follicular Fluid			Serum			Follicular Fluid			Serum			Follicular Fluid			Serum		
Fixed effect	F-value	df	*p*-value	F-value	df	*p*-value	F-value	df	*p*-value	F-value	df	*p*-value	F-value	df	*p*-value	F-value	df	*p*-value
Replicate tube	10.895	1	0.001	34.155	1	0.028	11.016	1	0.001	0.081	1	0.803	80.422	1	<0.001	45.576	1	<0.001
Random effects:	χ2	df	*p*-value	χ2	df	*p*-value	χ2	df	*p*-value	χ2	df	*p*-value	χ2	df	*p*-value	χ2	df	*p*-value
1|Male	128.587	1	<0.001	131.190	1	<0.001	72.455	1	<0.001	131.285	1	<0.001	64.600	1	<0.001	41.918	1	<0.001
1|Female	8.098	1	0.004	13.668	1	<0.001	0.350	1	0.554	6.895	1	0.009	0.000	1	1.000	0.000	1	1.000
1|Male:Female	0.827	1	0.363	0.535	1	0.465	11.528	1	<0.001	2.631	1	0.105	2.023	1	0.155	47.970	1	<0.001
1|Sample	0.000	1	1.000	0.352	1	0.553	0.000	1	1.000	7.043	1	0.008	0.000	1	1.000	0.000	1	1.000
	R^2^_m_ = 0.006; R^2^_c_ = 0.875			R^2^_m_ = 0.023; R^2^_c_ = 0.883			R^2^_m_ = 0.011; R^2^_c_ = 0.786			R^2^_m_ = 0.000; R^2^_c_ = 0.898			R^2^_m_ = 0.093; R^2^_c_ = 0.744			R^2^_m_ = 0.044; R^2^_c_ = 0.744		

**Table 2:** Time point-specific linear mixed models (LMM) testing for the association between sperm swimming velocity (VCL) in follicular fluid (response variable) and serum. Models included VCL in serum treatment and replicate tube as fixed effects and male, female, male:female interaction, and repeated measurement of each tube (sample) as random effects. R^2^m: marginal R^2^—proportion of variance explained by fixed factors; R^2^c: conditional R^2^—proportion of variance explained by fixed and random factors.

Time Point	60 Min			180 Min			300 Min		
Fixed Effects:	F-value	df	*p*-value	F-value	df	*p*-value	F-value	df	*p*-value
VCL in Serum	5.391	1	0.021	9.402	1	0.003	9.804	1	0.002
Replicate Tube	3.668	1	0.057	18.692	1	0.043	71.175	1	<0.001
Random Effects:	χ2	df	*p*-value	χ2	df	*p*-value	χ2	df	*p*-value
1|Male	84.694	1	<0.001	24.468	1	<0.001	30.020	1	<0.001
1|Female	5.978	1	0.014	0.524	1	0.469	0.0000	1	0.999
1|Male:Female	1.579	1	0.209	25.336	1	<0.001	8.6606	1	0.003
1|Sample	0.000	1	1.000	0.004	1	0.951	0.0000	1	0.999
	R^2^_m_ = 0.034; R^2^_c_ = 0.846			R^2^_m_ = 0.103; R^2^_c_ = 0.786			R^2^_m_ = 0.158; R^2^_c_ = 0.775		

**Table 3:** Linear mixed model (LMM) for sperm swimming velocity (VCL) and generalized mixed model (GLMM) for the proportion of hyperactivated sperm cells, testing for the consistency of male–female compatibility effect in different sperm treatments (180 min in follicular fluid vs. 300 min in serum, see Results). Models included treatment and replicate tube as fixed effects and interactions of treatment:male, treatment:female, treatment:male:female, and repeated measurement of each tube (sample) as random effects. The GLMM used a beta binomial distribution. R^2^m: marginal R^2^—proportion of variance explained by fixed factors; R^2^c: conditional R^2^—proportion of variance explained by fixed and random factors.

Response Variable	VCL			Hyperactivation		
Fixed Effects:	F-value	df	*p*-value	χ2	df	*p*-value
Treatment	2.143	1	0.160	1.870	1	0.171
Replicate Tube	44.988	1	<0.001	59.141	1	<0.001
Random Effects:	χ2	df	*p*-value	χ2	df	*p*-value
1|Treatment:Male	118.825	1	<0.001	124.81	1	<0.001
1|Treatment:Female	0.035	1	0.851	0.000	1	1.000
1|Treatment:Male:Female	49.487	1	<0.001	44.413	1	<0.001
1|Sample	0.000	1	1.000	0.000	1	1.000
	R^2^_m_ = 0.088; R^2^_c_ = 0.797			R^2^_m_ = 0.040; R^2^_c_ = 0.384		

## Data Availability

The data supporting the results is archived in the GitHub Repository and available at: https://github.com/alekslukasiewicz/Sperm_Physiological_Response_to_Female_Serum.git (accessed on 1 February 2022).

## Supplementary Materials

The following supporting information can be downloaded at: https://www.mdpi.com/article/10.3390/ijms23073428/s1.
