# Supplementary material for: Sperm Physiological Response to Female Serum—Potential New Insights into the Reproductive Incompatibility Diagnostics
Source: Int J Mol Sci. 2022 Mar 22;23(7):3428. doi: 10.3390/ijms23073428 (PMC8998597; doi:10.3390/ijms23073428)
Supplement: Supplementary file 1 [file ijms-23-03428-s001.zip › ijms-1603084-supplementary.pdf]

Supplementary Materials

Sperm physiological response to female serum – Potential new insights into the reproductive incompatibility diagnostics

Aleksandra Łukasiewicz[1*], Kari Huhta[1], Jarmo Ritari[2], Juha Peräsaari[3], Pia Allinen[4], Marjo Malinen[1], Annalaura Jokiniemi[1], Tanja Turunen[1], Jukka Partanen[2], Jukka Kekäläinen[1]

[1] Department of Environmental and Biological Sciences, University of Eastern Finland, P.O. Box 111, 80101 Joensuu, Finland;
[2] Research and Development, Finnish Red Cross Blood Service, Haartmaninkatu 8, 00290 Helsinki, Finland;
[3] Clinical Laboratory, Finnish Red Cross Blood Service, Kivihaantie 7, 00310 Helsinki, Finland;
[4] InOva klinikka Oy, Ajurinkatu 16, 70110 Kuopio, Finland;
[*] Correspondence: aleks.lukasiewicz@gmail.com

Table S1. Linear mixed model (LMM) for sperm swimming velocity (VCL) and generalized mixed model (GLMM) for proportion of hyperactivated sperm cells. Models included treatment, time point, treatment-timepoint interaction and replicate tube as fixed effects and random slope of timepoint for all random effects, i.e. two- and three-level interactions between sperm treatment and male, female, and male-female combination, and for sample (repeated measures of each tube). Full model for hyperactivation data was implemented with observational-level random effect (OLRE) to account for overdispersion.

Response variable	*VCL*			*Hyperactivation*		
Fixed effects:	***F-value***	***df***	***p-value***	***χ2***	***df***	***p-value***
Treatment	0.123	1	0.730	0.597	1	0.440
Time point	16.001	2	**<0.001**	32.844	2	**<0.001**
Treatment:Timepoint	4.461	2	**0.025**	15.001	2	**<0.001**
Replicate tube	17.187	1	**0.030**	1.513	1	0.219
Random effects:	***χ2***	***df***	***p-value***	***χ2***	***df***	***p-value***
Timepoint|Treatment:Male	135.784	5	**<0.001**	349.28	6	**<0.001**
Timepoint|Treatment:Female	12.013	5	**0.035**	29.233	6	**<0.001**
Timepoint|Treatment:Male:Female	**44.487**	5	**<0.001**	117.83	6	**<0.001**
Timepoint|Sample	137.214	5	**<0.001**	221.19	6	**<0.001**
1|OLRE	-	-	-	428.36	1	**<0.001**

Table S2. Time point-specific linear mixed models (LMM) for sperm swimming velocity (VCL). Models included treatment and replicate tube as fixed effects and interactions of treatment-male, treatment-female, treatment-male-female, and repeated measurement of each tube (sample) variable as random effects.

Time point	*60 minutes*			*180 minutes*			*300 min*		
Fixed effects:	***F-value***	***df***	***p-value***	***F-value***	***df***	***p-value***	***F-value***	***df***	***p-value***
Treatment	0.426	1	0.522	0.148	1	0.705	2.702	1	0.117
Replicate tube	49.784	1	**<0.001**	4.772	1	0.159	123.717	1	**<0.001**
Random effects:	***χ2***	***df***	***p-value***	***χ2***	***df***	***p-value***	***χ2***	***df***	***p-value***
1|Treatment:Male	258.986	1	**<0.001**	176.603	1	**<0.001**	100.117	1	**<0.001**
1|Treatment:Female	19.835	1	**<0.001**	2.424	1	0.1195	0.000	1	1.000
1|Treatment:Male:Female	1.345	1	0.2462	18.372	1	**<0.001**	45.698	1	**<0.001**
1|Sample	0.000	1	0.9998	0.768	1	0.3808	0.000	1	1.000

Table S3. Time point-specific generalized mixed models (GLMM) for proportion of hyperactivated sperm cells. Models included treatment and replicate tube as fixed effects and interactions of treatment-male, treatment-female, treatment-male-female, and repeated measurement of each tube (sample) as random effects. Models were implemented with observation-level random effect (OLRE, 180min and 300min) or with a beta-binomial error distribution (60min) to account for overdispersion.

Time point	*60 minutes*			*180 minutes*			*300 minutes*		
Fixed effects:	***χ2***	***df***	***p-value***	***χ2***	***df***	***p-value***	***χ2***	***df***	***p-value***
Treatment	0.635	1	0.426	0.191	1	0.662	1.798	1	0.180
Replicate tube	29.636	1	**<0.001**	7.596	1	**0.006**	147.017	1	**<0.001**
Random effects:	***χ2***	***df***	***p-value***	***χ2***	***df***	***p-value***	***χ2***	***df***	***p-value***
1|Treatment:Male	246.44	1	**<0.001**	188.59	1	**<0.001**	101.86	1	**<0.001**
1|Treatment:Female	24.515	1	**<0.001**	5.101	1	**0.024**	0.000	1	0.999
1|Treatment:Male:Female	1.456	1	0.228	13.182	1	**<0.001**	24.641	1	**<0.001**
1|Sample	0.236	1	0.627	0.382	1	0.537	0.000	1	0.999
1|OLRE	-	-	-	128.99	1	**<0.001**	379.63	1	**<0.001**

Table S4. Treatment-specific generalized mixed models (GLMM) for proportion of hyperactivated sperm cells in each of the three time points. Models included replicate tube as fixed effect and male, female, male-female interaction, and repeated measurement of each tube (sample) as random effects. Models were implemented with observation-level random effect (OLRE, 300min) or with a beta-binomial error distribution (60min and 180min) to account for overdispersion.

Time point	*60 minutes*						*180 minutes*						*300 minutes*					
Treatment	*Follicular fluid*			*Serum*			*Follicular fluid*			*Serum*			*Follicular fluid*			*Serum*		
Fixed effect	***χ2***	***df***	***p-value***	***χ2***	***df***	***p-value***	***χ2***	***df***	***p-value***	***χ2***	***df***	***p-value***	***χ2***	***df***	***p-value***	***χ2***	***df***	***p-value***
Replicate tube	10.871	1	**<0.001**	36.049	1	**<0.001**	20.947	1	**<0.001**	0.041	1	0.840	131.03	1	**<0.001**	46.776	1	**<0.001**
Random effects:	***χ2***	***df***	***p-value***	***χ2***	***df***	***p-value***	***χ2***	***df***	***p-value***	***χ2***	***df***	***p-value***	***χ2***	***df***	***p-value***	***χ2***	***df***	***p-value***
1|Male	140.49	1	**<0.001**	109.54	1	**<0.001**	78.189	1	**<0.001**	133.25	1	**<0.001**	50.984	1	**<0.001**	55.663	1	**<0.001**
1|Female	15.073	1	**<0.001**	10.307	1	**0.001**	0.045	1	0.831	16.779	1	**<0.001**	0.597	1	0.440	0.000	1	0.999
1|Male:Female	2.348	1	0.126	0.464	1	0.496	19.468	1	**<0.001**	0.000	1	1.000	1.59	1	0.207	20.574	1	**<0.001**
1|Sample	0.000	1	1.000	0.000	1	1.000	0.000	1	1.000	2.171	1	0.141	0.000	1	1.000	0.000	1	1.000
1|OLRE	-	-	-	-	-	-	-	-	-	-	-	-	-	-	-	269.95	1	**<0.001**

Table S5. Generalized mixed model (GLMM) for sperm viability. The model included treatment and replicate tube as fixed effects and interactions of treatment-male, treatment-female, treatment-male-female, and repeated measurement of each tube (sample) as random effects. The model was implemented with a beta-binomial error distribution to account for overdispersion.

Fixed effects:	***χ2***	***df***	***p-value***
Treatment	5.044	1	**0.025**
Replicate tube	19.478	1	**<0.001**

Random effects:	***χ2***	***df***	***p-value***
1|Treatment:Male	32.297	1	**<0.001**
1|Treatment:Female	1.5713	1	0.210
1|Treatment:Male:Female	30.083	1	**<0.001**
1|Sample	0.587	1	0.444

Table S6. Treatment-specific generalized mixed models (GLMM) for sperm viability. Models included replicate tube as fixed effect and male, female, male-female interaction, and repeated measurement of each tube (sample) as random effects. Models were implemented with a beta-binomial error distribution to account for overdispersion.

Treatment	*Follicular fluid*			*Serum*		
Fixed effect	***χ2***	***df***	***p-value***	***χ2***	***df***	***p-value***
Replicate tube	11.034		**<0.001**	26.213	1	**<0.001**
Random effects:	***χ2***	***df***	***p-value***	***χ2***	***df***	***p-value***
1|Male	18.391	1	**<0.001**	14.462	1	**<0.001**
1|Female	0.000	1	0.999	8.6278	1	**0.003**
1|Male:Female	23.07	1	**<0.001**	2.7776	1	0.096
1|Sample	0.000	1	0.999	0.000	1	0.999

Table S7. Time point-specific generalized mixed models (GLMM) testing for the association between proportion of hyperactivated sperm cells in follicular fluid (response variable) and serum. Models included proportion of hyperactivated sperm cells in serum treatment and replicate tube as fixed effects and male, female and male-female interaction, and repeated measurement of each tube (sample) as random effects. Models were implemented with a beta-binomial error distribution to account for overdispersion.

Time point	*60 minutes*			*180 minutes*			*300 minutes*		
Fixed effects:	***χ2***	***df***	***p-value***	***χ2***	***df***	***p-value***	***χ2***	***df***	***p-value***
Hyperactivation in serum	3.388	1	0.066	5.802	1	**0.016**	13.225	1	**<0.001**
Replicate tube	4.993	1	**0.025**	18.559	1	**<0.001**	81.954	1	**<0.001**
Random effects:	*χ2*	***df***	***p-value***	*χ2*	***df***	***p-value***	*χ2*	***df***	***p-value***
1|Male	134.430	1	**<0.001**	53.532	1	**<0.001**	42.014	1	**<0.001**
1|Female	13.340	1	**<0.001**	0.1304	1	0.718	1.807	1	0.179
1|Male:Female	2.227	1	0.136	16.928	1	**<0.001**	1.804	1	0.179
1|Sample	0.000	1	1.000	0.000	1	1.000	0.000	1	1.000

Table S8. Generalized mixed models (GLMM) testing for the association between sperm viability in follicular fluid (response variable) and serum. The model included proportion of dead sperm cells in serum treatment and replicate tube as fixed effects and male, female, male-female interaction, and repeated measurement of each tube (sample) as random effects. The model was implemented with a beta-binomial error distribution to account for overdispersion.

Fixed effects:	*$\chi 2$*	***df***	***p-value***
Viability in serum	5.696	1	**0.017**
Replicate tube	5.798	1	**0.016**

Random effects:	*$\chi 2$*	***df***	***p-value***
1|Male	12.319	1	**<0.001**
1|Female	0.000	1	0.999
1|Male:Female	26.207	1	**<0.001**
1|Sample	0.000	1	0.999

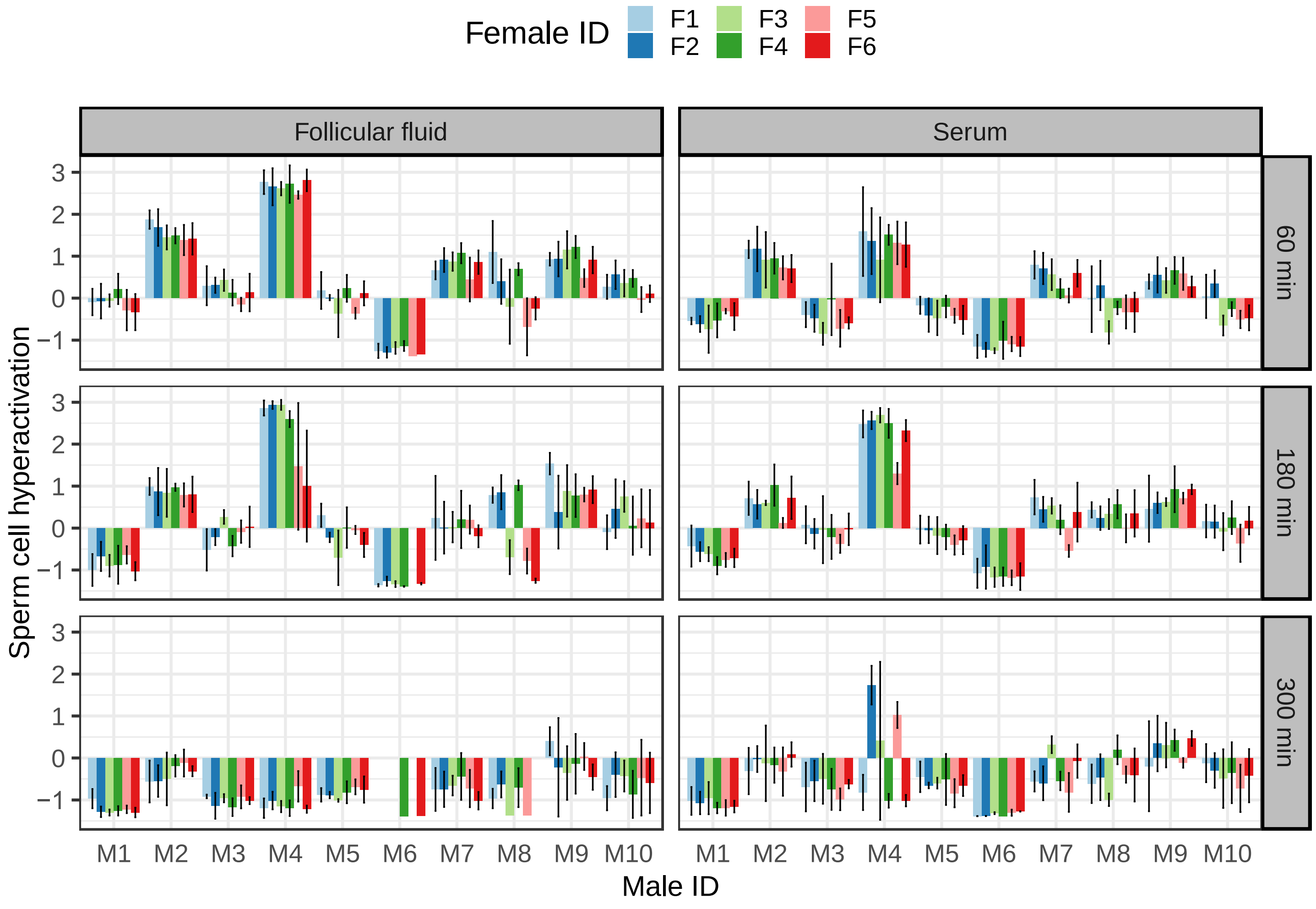

Figure S1. The effect of male–female interaction (combination) on the proportion of hyperactivated sperm cells (calculated on four replicate measurements per combination ± s.e.) at three time points (60, 180, and 300min) after the initiation of the follicular fluid or serum. The data were standardized to a mean of 0 with a standard deviation of 1.

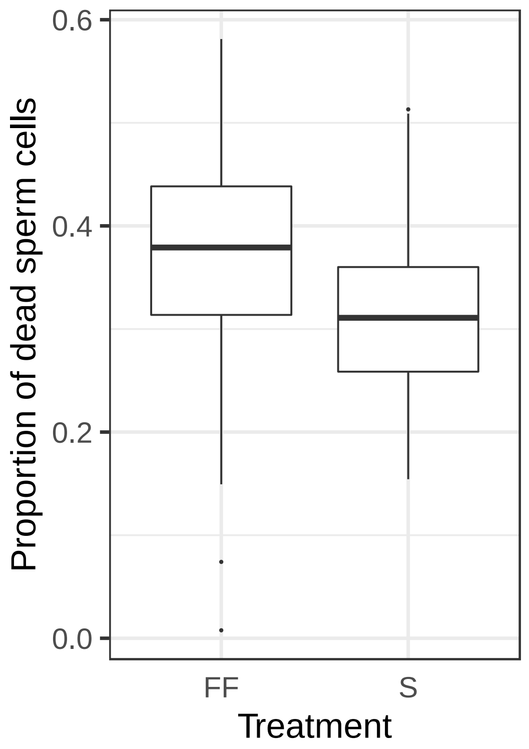

Figure S2. The effect follicular fluid (FF) and serum (S) treatment on the viability of sperm cells. The box encloses values between the first and third quartiles of the data (the inter-quartile range, IQR), while the horizontal bar within the box indicates the median. Whiskers extend from the box to the largest/smallest values that are within 1.5× the IQR of the box.

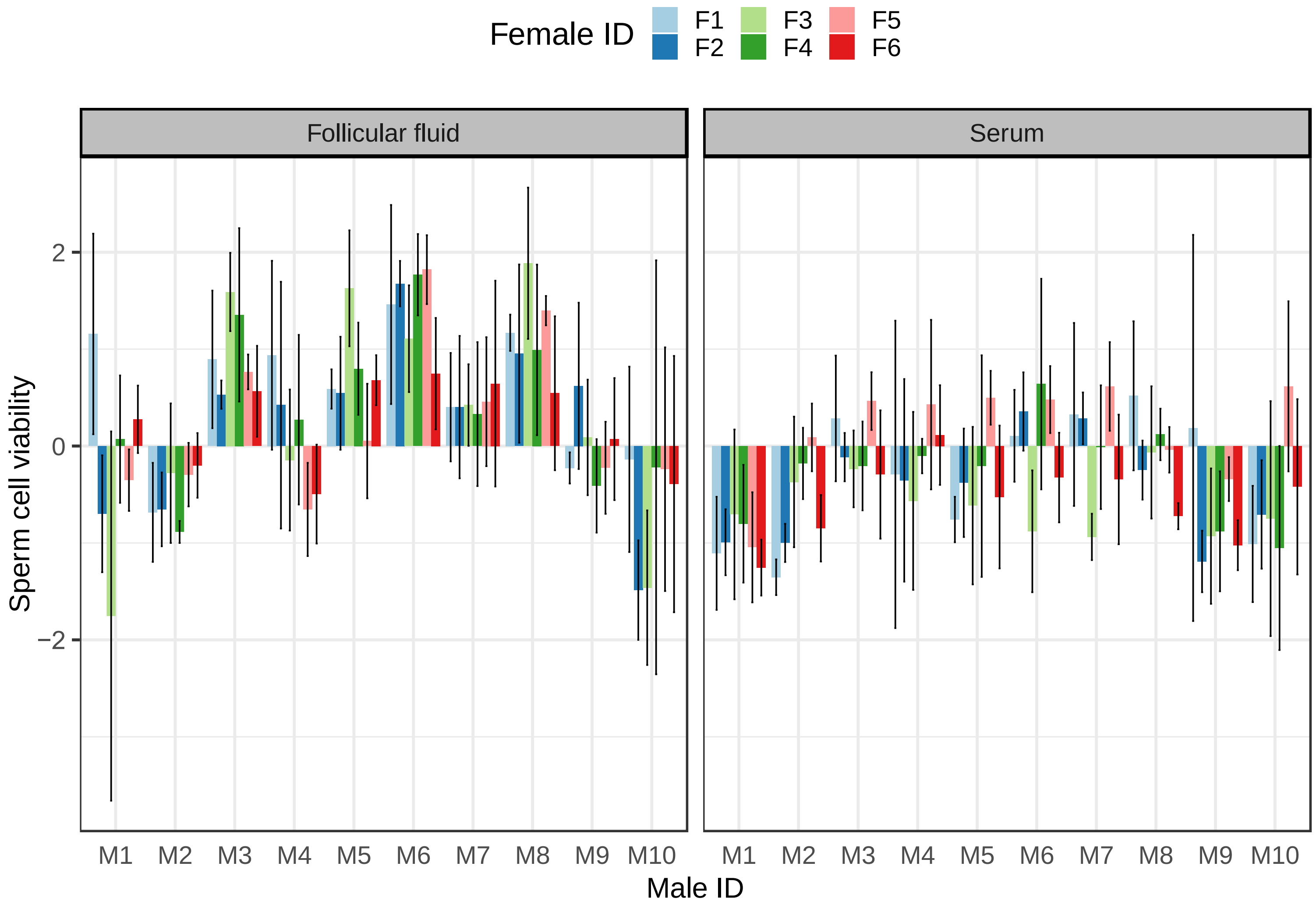

Figure S3. The effect of male–female interaction (combination) on sperm viability (calculated on two replicate measurements per combination ± s.e.). The data were standardized to a mean of 0 with a standard deviation of 1.
